# COVID-19 and cancer risk arising from ionizing radiation exposure through CT scans: a cross-sectional study

**DOI:** 10.1186/s12885-024-12050-x

**Published:** 2024-03-05

**Authors:** Golshan Mahmoudi, Heidar Toolee, Reza Maskani, Farzaneh Jokar, Milad Mokfi, Ali Hosseinzadeh

**Affiliations:** 1 School of Allied Medical Sciences, Shahroud University of Medical Sciences, Shahroud, Iran; 2 School of Medicine, Shahroud University of Medical Sciences, Shahroud, Iran; 3 Center for Health Related Social and Behavioral Sciences Research, Shahroud University of Medical Sciences, Shahroud, Iran; 4 Department of Epidemiology, School of Public Health, Shahroud University of Medical Sciences, Shahroud, Iran

**Keywords:** Radiation cancer risk, Computed tomography, COVID-19

## Abstract

**Background:**

The surge in the utilization of CT scans for COVID-19 diagnosis and monitoring during the pandemic is undeniable. This increase has brought to the forefront concerns about the potential long-term health consequences, especially radiation-induced cancer risk. This study aimed to quantify the potential cancer risk associated with CT scans performed for COVID-19 detection.

**Methods:**

In this cross-sectional study data from a total of 561 patients, who were referred to the radiology center at Imam Hossein Hospital in Shahroud, was collected. CT scan reports were categorized into three groups based on the radiologist’s interpretation. The BEIR VII model was employed to estimate the risk of radiation-induced cancer.

**Results:**

Among the 561 patients, 299 (53.3%) were males and the average age of the patients was 49.61 ± 18.73 years. Of the CT scans, 408 (72.7%) were reported as normal. The average age of patients with normal, abnormal, and potentially abnormal CT scans was 47.57 ± 19.06, 54.80 ± 16.70, and 58.14 ± 16.60 years, respectively (p-value < 0.001). The average effective dose was 1.89 ± 0.21 mSv, with 1.76 ± 0.11 mSv for males and 2.05 ± 0.29 mSv for females (p-value < 0.001). The average risk of lung cancer was 3.84 ± 1.19 and 9.73 ± 3.27 cases per 100,000 patients for males and females, respectively. The average LAR for all cancer types was 10.30 ± 6.03 cases per 100,000 patients.

**Conclusions:**

This study highlights the critical issue of increased CT scan usage for COVID-19 diagnosis and the potential long-term consequences, especially the risk of cancer incidence. Healthcare policies should be prepared to address this potential rise in cancer incidence and the utilization of CT scans should be restricted to cases where laboratory tests are not readily available or when clinical symptoms are severe.

## Background

Cancer is a leading cause of death worldwide, with 19.3 million new cases, and nearly 10 million deaths worldwide in 2020 [1]. The incidence of cancer cases and mortality is expected to increase as populations grow, age, and adopt lifestyle choices that heighten cancer risk [2]. One of the risk factors that may increase an individual’s likelihood of developing cancer is exposure to ionizing radiation. Robust epidemiological evidence substantiates that exposure to low-level ionizing radiation at doses used in medical imaging can contribute to an increased risk of cancer incidence [3–6].

Presently, computed tomography (CT) is responsible for a substantial portion of medical radiation exposure, with over 80 million CT scans conducted annually in the United States [7, 8]. CT scans deliver a much higher radiation doses compared to conventional diagnostic X-rays, potentially amplifying the risk of cancer incidence [9]. It has been estimated that a CT scan with an effective dose of 10 mSv increases cancer incidence by approximately 1 in 2,000 [10]. While this may seem inconsequential on an individual level, within a large population, it becomes a significant concern. Consequently, it appears that CT scans may potentially contribute to cancer cases rather than preventing them, particularly as the use of repeated CT screening and follow-up scans rises [11].

The urgent need for rapid diagnosis during the COVID-19 pandemic has led to the continued utilization of CT examinations in numerous countries for early disease diagnosis and monitoring [12]. The real-time reverse transcription-polymerase chain reaction (RT-PCR), widely known as the gold standard for confirming COVID-19 diagnosis, exhibits variable and often limited sensitivity, ranging from 32 to 80% [13, 14]. Hence, CT scans, known for their high sensitivity (approximately 97%) and widespread availability, have become a widely adopted method for COVID-19 diagnosis [15, 16]. Nevertheless, this increased dependence on CT scans during the COVID-19 crisis has raised concerns about potential long-term health consequences, especially the risk of cancer incidence, such that several international radiology associations discourage the use of CT scans as a primary diagnostic tool [17, 18]. Therefore, despite the benefits of CT scans in early COVID-19 detection and management, comprehending the long-term implications of their increased usage is imperative. This understanding aids in striking a balance between potential harm and benefit. Furthermore, estimating cancer risk is pivotal for shaping healthcare policies and guiding clinical decision-making. To the best of our knowledge, the cancer risk associated with chest CT scans used for COVID-19 diagnosis in Iran remains inadequately understood. This study aimed to investigate the potential consequences of increased CT scan usage during the COVID-19 pandemic on future cancer incidence resulting from ionizing radiation exposure.

## Methods

### Study population and data collection

Data from 561 patients who had undergone chest CT scans for COVID-19 detection between November 19th and December 6th 2022, at the emergency radiology center of Imam Hossein Hospital in Shahroud, Iran, was collected. Patient data were extracted from their medical records, the picture archiving and communication system (PACS), and the CT scanner. This dataset includes demographic variables, including age, gender, insurance details, referral frequency, CT scan reports, acquisition, and exposure parameters, as well as dosimetry data, including the volumetric computed tomography dose index (CTDI_vol_), and dose length product (DLP). The CT scan reports were categorized into three groups based on the radiologist’s interpretation: score 0, indicating no pathological findings, representing a normal CT scan; score 1, indicating suspicious findings, representing a potentially abnormal CT scan; and score 2, confirming pathological findings, representing an abnormal CT scan. Abnormal findings refer to clear and confirmed pathological changes or abnormalities observed in the lung images that are indicative of COVID-19-related abnormalities. These findings suggest the presence of lung lesions or characteristics associated with the disease. Potentially abnormal findings imply suspicious observations that may indicate abnormalities, but they lack confirmation or clear evidence of pathology related to COVID-19. These findings raise concern but require further investigation or confirmation to establish a definitive link to the virus or other underlying conditions.

### CT equipment and technique

All CT scans were performed using a Siemens SOMATOM Emotion 6-slice CT scanner (Siemens Healthcare, Germany). The Chest protocols entailed 130 kVp, effective mAs was 27, pitch 1.4, and 4 mm slice thickness.

### Cancer risk estimation

Lifetime attributable risk (LAR) represents the probability of prematurely developing cancer due to radiation exposure in the population. LAR is contingent on gender, age at exposure, and the specific organ under consideration. In this study, LARs for cancer incidence were estimated using the Biological Effects of Ionizing Radiations (BEIR) VII models [19]. BEIR VII models employ organ doses to estimate cancer incidence based on gender and age at exposure. It is expressed as the incidence of cancers per 100,000 individuals exposed to a single dose of 100 mSv. To calculate the radiation cancer risk, LARs were interpolated for the study population (both males and females), and the risk of cancer per 100,000 individuals was determined using the following formula:

1 $$LAR=LA{R_{100}} \times \frac{H}{{100}}$$

where LAR_100_ is the BEIR VII risk estimate, and H is the equivalent dose to the organ.

The equivalent doses to the organ were calculated using VirtualDose™ CT software [20]. VirtualDose is a web-based dose calculator software equipped with 25 phantoms, incorporating the latest CT scanners, and adhering to the most recent recommendations from ICRP-60 and ICRP-103. It facilitates the online calculation of patients’ doses, allowing users to evaluate organ dose and effective dose. The equivalent doses to the following organs were reported: stomach, colon, liver, lung, bladder, thyroid, prostate, breast, and uterus.

### Statistical analysis

The dataset was summarized using descriptive statistics, presenting continuous data as mean ± standard deviation and categorical data as numerical values (percentages). IBM SPSS version 21 (IBM Corporation, Armonk, New York, USA) was utilized for statistical analysis. Independent t-tests, Chi-square tests, one-way ANOVA, and Mann-Whitney U tests were employed to assess continuous and categorical data. Statistical significance was established at a p-value less than 0.05.

## Results

This study included a total of 561 examined patients, with 299 (53.3%) males and 262 (46.7%) females. Their ages ranged from 5 to 93 years, with an average age of 49.61 ± 18.73. Specifically, the mean age for males and females was 48.80 ± 17.97 and 50.55 ± 19.56 years, respectively. The demographic data and age groups according to the BEIR VII report have been summarized in Table 1.

**Table 1:** Number of patients which belonged to each age group according to the BEIR VII report

age group (y)	female	male	both gender
2.5–7.5	1	0	1
7.5–12.5	2	0	2
12.5–17.5	4	10	14
17.5–25	24	20	44
25–35	36	50	86
35–45	43	58	101
45–55	45	47	92
55–65	35	50	85
65–75	41	36	77
> 75	31	28	59
Total	262 (46.7%)	299 (53.3%)	561 (100%)

Of 561 examined patients 408 (72.7%) patients had normal CT scan reports, 14 (2.5%) had potentially abnormal reports, and the remaining 139 (24. 8%) had abnormal reports. The most common chest CT findings in COVID-19 were ground-glass opacities (GGOs), consolidation, a crazy-paving pattern, interlobular septal thickening, air bronchogram, and vascular enlargement which often involve both lungs with the peripheral distribution. Among those with normal reports, 220 (53.9%) were males, and 188 (46.1%) were females. In the group with abnormal reports, 76 (54.7%) were males, and 63 (45.3%) were females. Therefore, males exhibited a higher count of both normal and abnormal CT scans compared to females, and these differences were statistically significant (p-value < 0.05) as determined by the Chi-square test (Table 2).

**Table 2:** Frequency and percentages of normal, abnormal, and potentially abnormal CT scan reports

Gender	CT report		
	normal	abnormal	potentially abnormal
Male Female Total	220 (53.9%) 188 (46.1%) 408 (72.7%)	76 (54.7%) 63 (45.3%) 139 (24.8%)	3 (21.4%) 11 (78.6%) 14 (2.5%)

**Fig. 1:**
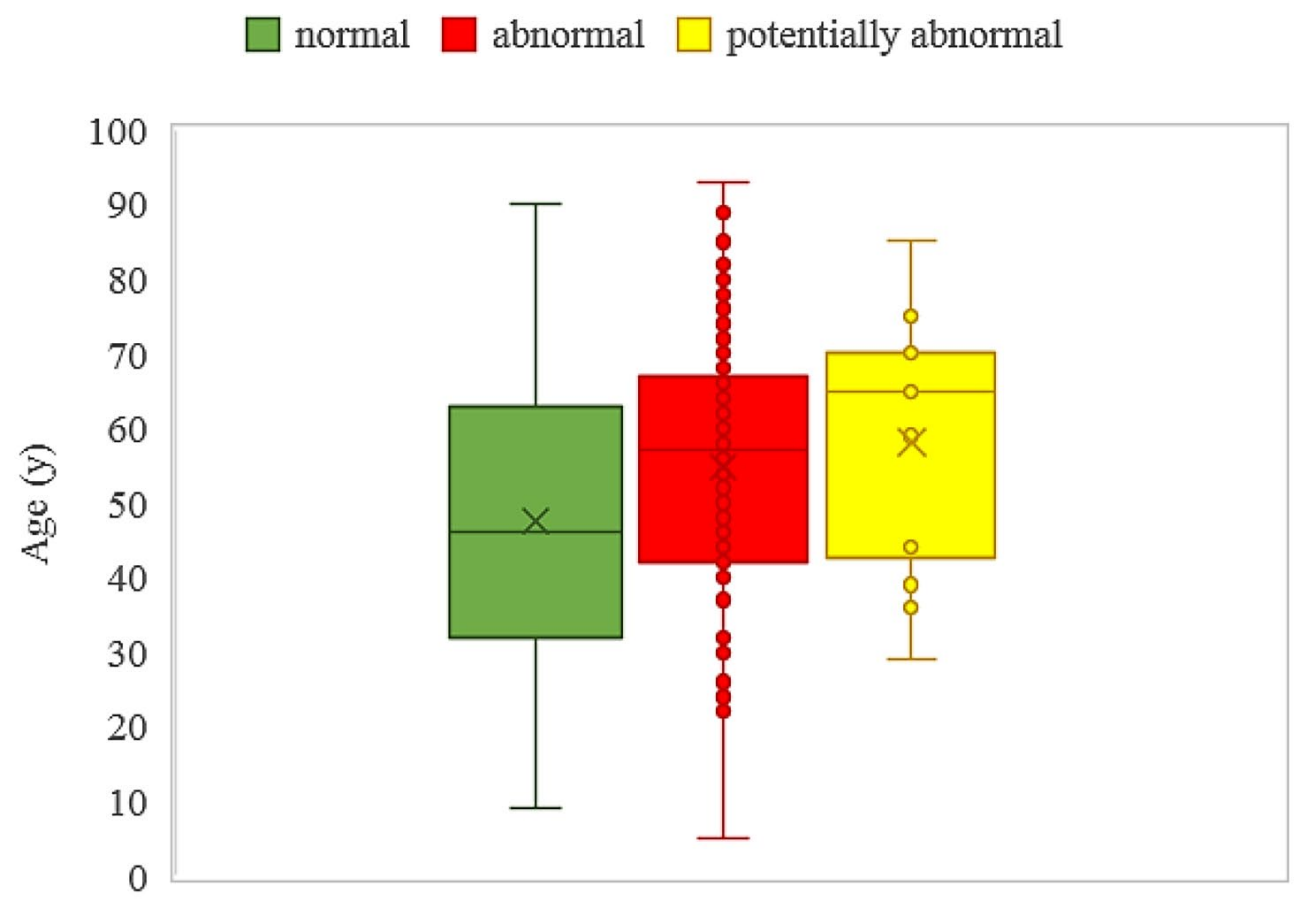
Age distribution concerning the normal, abnormal, and potentially abnormal CT scan report

The average ages of patients with normal, abnormal, and potentially abnormal CT scans were 47.57 ± 19.06, 54.80 ± 16.70, and 58.14 ± 16.60 years, respectively. These age differences were statistically significant according to one-way ANOVA (p-value < 0.001) (Fig. 1).

**Fig. 2:**
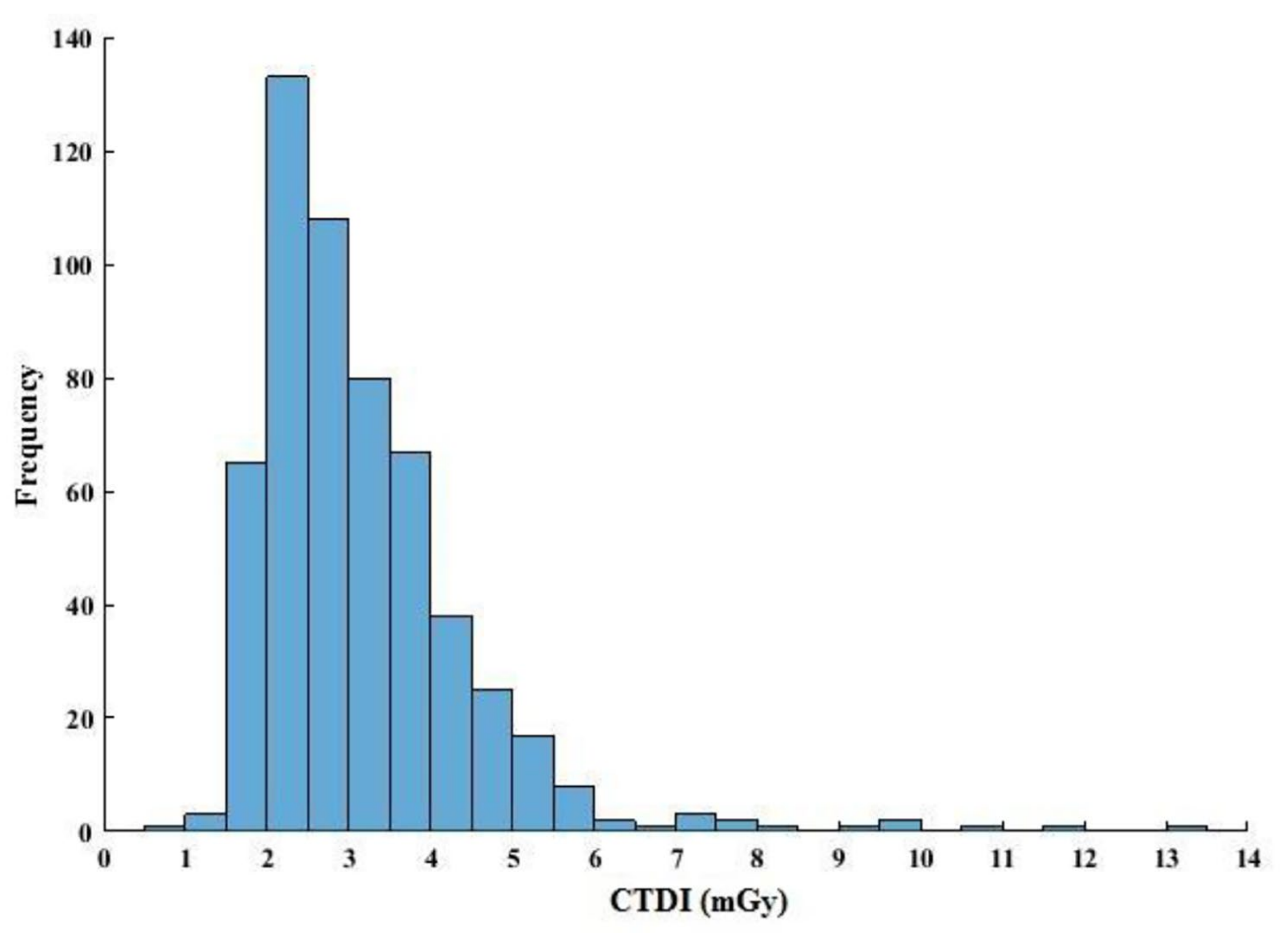
CTDI_vol_ (in mGy) frequency distribution

**Fig. 3:**
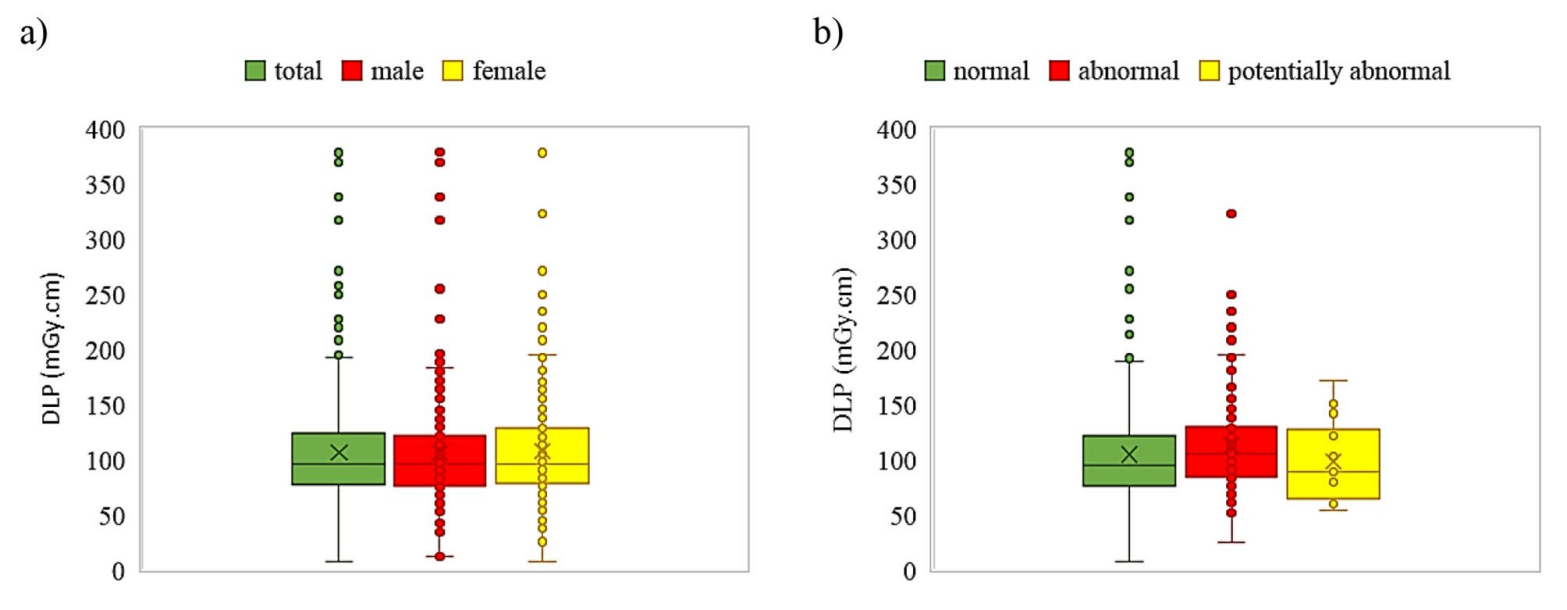
The box plot of DLP in mGy.cm, for (**a**) males, females, and total cases and (**b**) normal, abnormal, and potentially abnormal CT report

The frequency distribution of CTDI_vol_ is presented in Fig. 2, and corresponding CTDI_vol_ and DLP administered to each age group, for both males and females are detailed in Table 3. The average CTDI_vol_ was 3.33 ± 4.61 mGy, with values of 3.02 ± 1.18 mGy for males and 3.70 ± 6.63 mGy for females. The mean value of DLP was 106.33 ± 45.93 mGy.cm, comprising 107.04 ± 43.63 mGy.cm for males and 105.50 ± 48.51 mGy.cm for females (Fig. 3.a). There was no significant difference between males and females concerning the administered dose, as determined by an independent t-test (p-value < 0.97). Additionally, there was no correlation between DLP and age (*r* = 0.03, p-value < 0.52). Figure 3.b shows the box plot of DLP for different CT reports. The average DLP for the normal, abnormal, and potentially abnormal groups was 104.56 ± 46.98, 112.33 ± 43.31, and 97.89 ± 36.49, respectively, and one-way ANOVA indicated that these differences were not statistically significant (p-value < 0.17).

**Table 3:** The average ± standard deviation for CTDI_vol_, and DLP at each age group for males, females, and for total population

Age group	CTDI_vol_ (mGy)		DLP (mGy.cm)	
	male	female	male	female
2.5–7.5	-	2.70	-	25.20
7.5–12.5	-	1.67 ± 0.29	-	88.29 ± 47.18
12.5–17.5	2.46 ± 0.91	1.91 ± 0.23	89.27 ± 42.98	62.24 ± 7.17
17.5–25	2.84 ± 0.76	2.41 ± 0.70	99.16 ± 28.00	76.61 ± 28.37
25–35	3.12 ± 1.10	3.35 ± 1.80	109.48 ± 40.09	104.74 ± 56.88
35–45	3.22 ± 1.40	3.50 ± 2.11	115.26 ± 50.75	106.98 ± 54.86
45–55	3.05 ± 1.51	3.74 ± 1.06	109.51 ± 57.30	123.29 ± 38.11
55–65	3.03 ± 1.00	3.35 ± 1.02	106.53 ± 33.98	107.96 ± 34.09
65–75	3.07 ± 1.12	3.60 ± 1.76	108.01 ± 42.16	120.62 ± 61.01
> 75	2.63 ± 0.82	2.81 ± 0.77	93.16 ± 30.11	86.91 ± 31.62
Total	3.02 ± 1.18	3.70 ± 6.63	107.04 ± 43.63	105.50 ± 48.51
	3.33 ± 4.61		106.33 ± 45.93	

Among males, the lung received the highest equivalent dose (4.00 ± 0.22 mSv). In females, the highest equivalent doses were recorded for the lung (4.58 ± 0.60 mSv) and breast (4.06 ± 0.54 mSv). The average effective dose was 1.89 ± 0.21 mSv with 1.76 ± 0.11 mSv for males and 2.05 ± 0.29 mSv for females. There was a significant difference between males and females (p-value < 0.001) (Table 4).

**Table 4:** Equivalent organ dose for males and females

Organ	Equivalent organ dose (mSv)	
	male	female
Stomach	2.10 ± 0.22	2.59 ± 0.42
Colon	0.31 ± 0.05	0.39 ± 0.07
liver	2.35 ± 020	2.49 ± 0.35
lung	4.00 ± 0.22	4.58 ± 0.60
Bladder	0.05 ± 0.01	0.07 ± 0.01
Thyroid	0.75 ± 0.08	0.80 ± 0.10
prostate	0.04 ± 0.01	-
Breast	-	4.06 ± 0.54
uterus	-	0.14 ± 0.03
ED	1.76 ± 0.11	2.05 ± 0.29

**Fig. 4:**
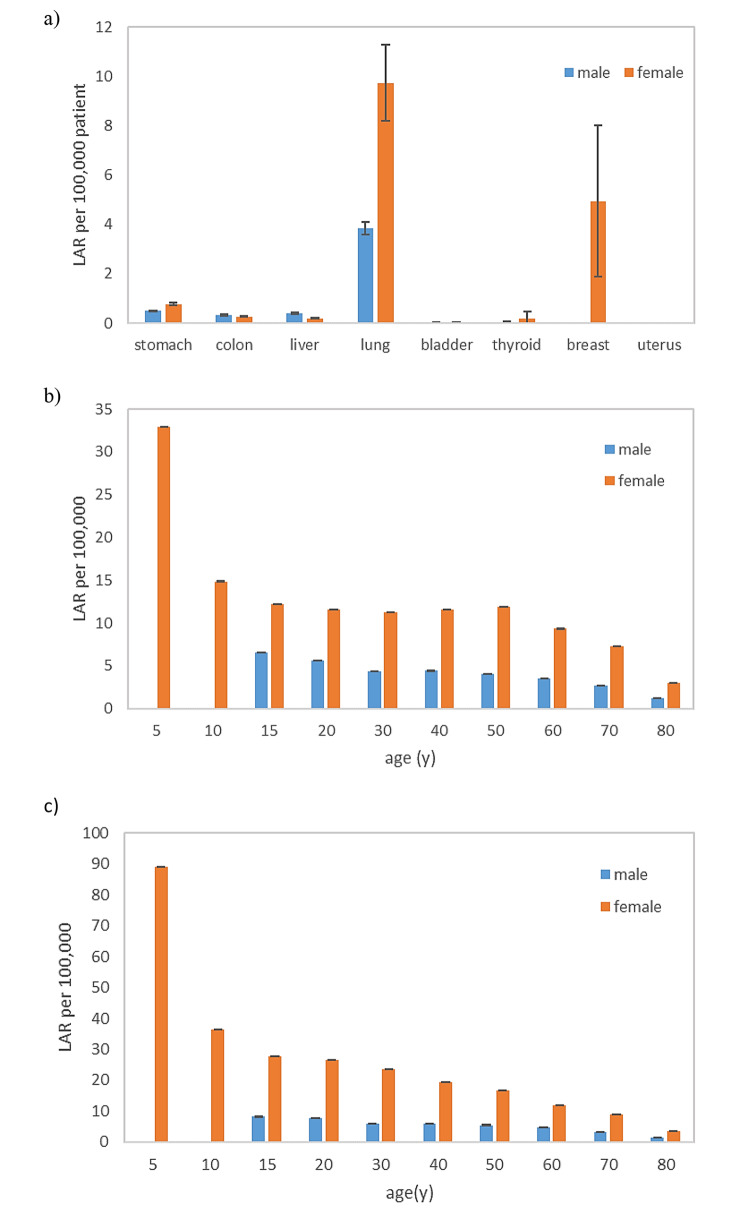
Lifetime attributable risk per 100,000 patients subjected to CT scan for COVID-19 diagnosis for (**a**) each cancer site for males and females, (**b**) lung cancer for different age groups, and (**c**) all cancer types considered in this study

The risk of lung cancer was higher in males (3.84 ± 1.19), while females exhibited significant risks for both lung (9.73 ± 3.27) and breast (4.95 ± 5.31) cancers (Fig. 4.a). The average LAR for all cancer types was 10.30 ± 6.03 per 100,000 patient. It was 5.16 ± 1.67 and 16.16 ± 8.64 per 100,000 patient in males and females, respectively. Consequently, the risk for both lung cancer (Fig. 4.b) and all cancer (Fig. 4.c) types is higher for females than for males.

The average referral frequency of the patients was 1.67 ± 2.87. This finding means that each patient has undergone a CT scan for COVID-19 diagnosis more than one time on average. Notably, 84.35% of the patients underwent a CT scan for the first time, with 71% of their reports being normal. Additionally, the average referral frequency for males and females was 1.60 ± 2.89 and 1.75 ± 2.84, respectively. The Mann-Whitney U test demonstrated that this difference was not statistically significant (p-value < 0.33).

## Discussion

We employed the BEIR VII model to estimate the cancer incidence risk associated with ionizing radiation exposure from CT scans used for diagnosing COVID-19. The average cancer incidence risk for all types of cancers considered in this study was approximately 11 cases per 100,000 patients, with 5.16 cases for males and 16.16 cases for females. This finding underscores the significant impact of radiation exposure from CT scans in diagnosing COVID-19 on the elevated cancer incidence in the population.

The cancer risk estimates in the present study were notably lower than those reported in similar studies. For instance, in our study, the estimated lung cancer risks for males and females were approximately 4 and 10 cases per 100,000 patients, respectively. In contrast, a study by Ghetti et al. reported lung cancer risks of approximately 10 and 30 cases per 100,000 patients for males and females, respectively [21]. These discrepancies were also evident in dosimetry data. The mean effective dose in our study was 1.89 ± 0.07 mSv, while Ghetti et al. reported a mean effective dose of 4.4 ± 1.6 mSv [21]. Additionally, the mean values of CTDI_vol_ and DLP in our study were 3.33 ± 4.61 mGy and 106.33 ± 45.93 mGy.cm, whereas Ghetti et al. reported values of 6.8 ± 2.7 mGy and 239 ± 94 mGy.cm [21]. This remarkable difference can be attributed to the different imaging protocols. The routine chest CT protocol had been optimized in our radiology center to reduce the radiation dose of COVID-19 patients without reducing its efficiency. Several studies confirmed the effectiveness of low-dose and ultra-low-dose CT scans in detecting and evaluating COVID-19 [22–25]. Therefore, in pandemics such as COVID-19 where ionizing radiation is employed for screening or follow-up of a large population, radiation settings can be significantly lower than in routine scans [26]. Deciding to reduce patient’s radiation dose is critical and can significantly affect the cumulative dose of patients and reduce the stochastic effect of radiation, including radiation-induced cancer [27].

The cancer risk estimates revealed a notable disparity between males and females, indicating that females face a substantially higher risk of radiation-induced cancer compared to males. The increased cancer incidence risk among females is attributed to their greater sensitivity to radiation. This risk is dependent on LAR and effective dose (Eq. 1). The LAR values of the BEIR VII report were higher for females than males [19]. Furthermore, the results indicated that females received a higher effective dose (2.05 ± 0.29 mSv) than males (1.76 ± 0.11 mSv), consistent with previous research [21, 28, 29].

The results demonstrated that the cancer risk decreased in both genders as the age at the time of exposure increased. Extensive evidence from survivors of the Japanese atomic bombings suggests that children are more vulnerable than adults to the adverse effects of ionizing radiation [30]. Younger individuals have a longer life expectancy following exposure to ionizing radiation, providing more time for cancer to develop [28]. Therefore, our study population, more than 50% of whom were under 50 years old, further increased cancer risk. Overall, considering both gender and age factors, younger females are at a higher risk of radiation-induced cancer, requiring fewer CT scans to result in cancer compared to other populations [28].

As expected, lung cancer was the main disease that could be induced by chest CT scans for both males (3.84 cases per 100,000 patients) and females (9.73 cases per 100,000 patients). Additionally, the risk of breast cancer was considerable for females (4.95 cases per 100,000 patients). This is because the lungs and breasts receive the highest equivalent dose in the chest CT scans for both genders (Table 4). This finding is consistent with previous studies that reported a higher risk of lung cancer for men and lung and breast cancer for women [21, 31]. In light of these findings, policymakers and public health leaders should emphasize screening programs, especially for lung and breast cancers, targeting high-risk groups in the future.

Altogether, it is crucial to recognize that even a slight increase in cancer risk at the population level can translate into a substantial number of additional cancer cases over time [4, 27]. For example, it is commonly reported that a CT scan may be associated with an increase in the risk of cancer of approximately 1 in 2,000 [10]. Until November 5th, 2023, 697,369,411 COVID-19 cases had been recorded and 669,159,941 of them have been recovered [32]. Considering a cancer risk of 1 in 2,000, there is a possibility of developing radiation-induced cancer in 334,580 cases. Since cancer risk increases with cumulative radiation dose, reducing the radiation dose delivered to the population in pandemic diagnostics could substantially decrease this excess cancer risk. Therefore, efforts should be made to minimize radiation exposure from CT scans. In this regard, several general approaches should be taken.

First, reducing the number of CT examinations. Although assessing this fully is challenging, a considerable percentage of the CT examinations currently conducted may be unnecessary [28]. For example, in this study, we found that a large percentage (72.7%) of CT scans performed for COVID-19 diagnosis were reported normal, indicating the absence of pathological findings related to COVID-19 and it is possible that many of them were unnecessary. The diagnostic value of CT scans needs to be balanced against the risk of carcinogenesis associated with their use. The threshold for using CT has lowered, and it is now employed not only in severely ill patients but also in individuals with mild, self-limited illnesses who are otherwise healthy [28]. One way to address this issue is to identify clinical factors that can predict the severity of the disease. For instance, our results showed that patients with normal CT scans were generally younger than those with abnormal or potentially abnormal CT scans, suggesting that older individuals may be more likely to have COVID-19, in line with previous studies [33, 34]. Several studies have shown the effectiveness of identifying clinical factors to reduce unnecessary CT scans [35, 36]. Consequently, it appears that during pandemics like COVID-19, radiological examinations should not be the first-line diagnostic tool as recommended by the American College of Radiology [37]. CT scans for COVID-19 diagnosis should be restricted to cases where laboratory tests are not readily available or when clinical symptoms are severe. Moreover, decreasing the number of CT scans performed for follow-up, especially for patients with improving symptoms could minimize unnecessary CT scans [38, 39].

Second, CT examination protocols and techniques should be optimized and standardized to minimize radiation exposure for individual scans, especially in the case of follow-up examinations, where patients are exposed multiple times within a short period. Due to the cumulative effects of radiation dose, radiation conditions should be kept as minimal as possible. Several studies have demonstrated dose reduction through optimized techniques for COVID-19 diagnosis by reducing mAs and increasing pitch and slice thickness [22–25]. Additionally, our study demonstrated that the radiation dose can be substantially decreased through the optimization of the imaging protocol. Consequently, international committees should provide new optimized imaging protocols for situations like the COVID-19 pandemic when radiation examination is widely used for screening and follow-up.

The third approach is raising awareness about the risks of ionizing radiation. Neither physicians, nor patients are generally aware of the radiation associated with CT, its risk of carcinogenesis, nor the importance of limiting exposure among younger patients [40–43]. Both physicians and patients need to be made aware of this risk. A study by Gimbel et al. demonstrated a significant decrease in CT scan orders after providing physicians with information about the health risks of ionizing radiation [44].

Our study has some limitations. One limitation is the use of a single CT scanner in a single hospital with a limited number of patients. Moreover, the BEIR VII model we employed in this study estimates cancer risk based on the linear no-threshold model, which some studies suggest overestimates radiation cancer risk at the low-dose radiation levels typically used in X-ray imaging [45, 46]. Despite these limitations, our study highlights the critical issue of increased CT scan usage for COVID-19 diagnosis and the potential long-term consequences, particularly in terms of future cancer incidence. As a result, healthcare policies should be prepared to address the potential rise in cancer incidence due to the increased use of CT scans for COVID-19 diagnosis. This research not only underscores the risks associated with CT scans during the COVID-19 pandemic but also underscores the importance of continued vigilance in healthcare practice. The choice of diagnostic methods must be carefully considered in light of the immediate clinical need to diagnose and manage patients, especially during pandemics. Understanding the interplay between the risks and benefits of medical interventions like CT scans is essential for informed healthcare policies and clinical guidelines. It is vital to maintain a balanced perspective when weighing the immediate benefits of CT scans against the potential future health risks they may pose, especially when applied to populations with varying vulnerabilities and in different clinical scenarios.

## Conclusions

This study highlights the potential for increased cancer incidence in the years to come due to the escalated use of CT scans during the COVID-19 pandemic. Therefore, healthcare policies should be prepared to address this potential rise in cancer incidence. This underscores the importance of adopting a nuanced approach to diagnostic and screening procedures. The use of CT scans should be limited to cases where laboratory tests are not readily available or when clinical symptoms are severe. It is vital to maintain a balanced perspective when weighing the immediate benefits of CT scans against the potential future health risks they may pose, especially when applied to populations with different vulnerabilities and across various clinical scenarios.

## Data Availability

Access to detailed individual data can be obtained from the corresponding authors upon reasonable request.

## Abbreviations

CT: Computed tomography
RT-PCR: Real-time reverse transcription-polymerase chain reaction
PACS: Picture archiving and communication system
CTDI_vol_: Volumetric computed tomography dose index
DLP: Dose length product
LAR: Lifetime attributable risk
BEIR: Biological effects of ionizing radiations

## References

[1] Sung H, Ferlay J, Siegel RL, Laversanne M, Soerjomataram I, Jemal A (2021). Global Cancer statistics 2020: GLOBOCAN estimates of incidence and Mortality Worldwide for 36 cancers in 185 countries. Cancer J Clin.

[2] Torre LA, Siegel RL, Ward EM, Jemal A (2016). Global Cancer Incidence and Mortality Rates and Trends–An Update. Cancer epidemiology, biomarkers & prevention: a publication of the American Association for Cancer Research. Cosponsored Am Soc Prev Oncol.

[3] Berrington de Gonzalez A, Daniels RD, Cardis E, Cullings HM, Gilbert E, Hauptmann M (2020). Epidemiological studies of low-dose Ionizing Radiation and Cancer: Rationale and Framework for the Monograph and Overview of Eligible studies. J Natl Cancer Inst Monogr.

[4] Cao C-F, Ma K-L, Shan H, Liu T-F, Zhao S-Q, Wan Y (2022). CT scans and cancer risks: a systematic review and dose-response meta-analysis. BMC Cancer.

[5] Linet MS, Kim KP, Miller DL, Kleinerman RA, Simon SL, Berrington de Gonzalez A (2010). Historical review of occupational exposures and cancer risks in medical radiation workers. Radiat Res.

[6] Mathews JD, Forsythe AV, Brady Z, Butler MW, Goergen SK, Byrnes GB et al. Cancer risk in 680 000 people exposed to computed tomography scans in childhood or adolescence: data linkage study of 11 million australians. 2013;346:f2360. 10.1136/bmj.f2360 PMC3660619 23694687

[7] Mansour HH, Alajerami YS, Foster T. Estimation of Radiation doses and lifetime attributable risk of Radiation-induced Cancer from a single coronary artery bypass graft computed Tomography Angiography. J Electron J Gen Med. 2021;18(6).

[8] Schultz CH, Fairley R, Murphy LS, Doss M (2020). The risk of Cancer from CT scans and other sources of low-dose Radiation: a critical Appraisal of Methodologic Quality. Prehosp Disaster Med.

[9] Power SP, Moloney F, Twomey M, James K, O’Connor OJ, Maher MM (2016). Computed tomography and patient risk: facts, perceptions and uncertainties. World J Radiol.

[10] Berrington de González A, Mahesh M, Kim KP, Bhargavan M, Lewis R, Mettler F (2009). Projected cancer risks from computed tomographic scans performed in the United States in 2007. Arch Intern Med.

[11] Twombly R (2004). Full-body CT screening: preventing or producing cancer?. J Natl Cancer Inst.

[12] Peeling RW, Heymann DL, Teo YY, Garcia PJ (2022). Diagnostics for COVID-19: moving from pandemic response to control. Lancet (London England).

[13] Fang Y, Zhang H, Xie J, Lin M, Ying L, Pang P (2020). Sensitivity of chest CT for COVID-19: comparison to RT-PCR. Radiology.

[14] Yang W, Yan F (2020). Patients with RT-PCR-confirmed COVID-19 and normal chest CT. Radiology.

[15] Ai T, Yang Z, Hou H, Zhan C, Chen C, Lv W (2020). Correlation of chest CT and RT-PCR testing for Coronavirus Disease 2019 (COVID-19) in China: a report of 1014 cases. Radiology.

[16] Caruso D, Zerunian M, Polici M, Pucciarelli F, Polidori T, Rucci C (2020). Chest CT features of COVID-19 in Rome, Italy. Radiology.

[17] American College oR. ACR recommendations for the use of chest radiography and computed tomography (CT) for suspected COVID-19 infection [Available from: https://www.acr.org/Advocacy-and-Economics/ACR-Position-Statements/Recommendations-for-Chest-Radiography-and-CTfor-Suspected-COVID19-Infection.

[18] World Health O (2020). Use of chest imaging in COVID-19: a rapid advice guide, 11 June 2020.

[19] Council NR (2006). Health risks from exposure to low levels of Ionizing Radiation: BEIR VII phase 2.

[20] Ding A, Gao Y, Liu H, Caracappa PF, Long DJ, Bolch WE (2015). VirtualDose: a software for reporting organ doses from CT for adult and pediatric patients. Phys Med Biol.

[21] Ghetti C, Ortenzia O, Maddalo M, Altabella L, Sverzellati N (2020). Dosimetric and radiation cancer risk evaluation of high resolution thorax CT during COVID-19 outbreak. Physica Medica: PM: an international journal devoted to the applications of physics to medicine and biology. Official J Italian Association Biomedical Phys (AIFB).

[22] Tabatabaei SMH, Talari H, Gholamrezanezhad A, Farhood B, Rahimi H, Razzaghi R (2020). A low-dose chest CT protocol for the diagnosis of COVID-19 pneumonia: a prospective study. Emerg Radiol.

[23] Greffier J, Hoballah A, Sadate A, de Oliveira F, Claret PG, de Forges H (2021). Ultra-low-dose chest CT performance for the detection of viral pneumonia patterns during the COVID-19 outbreak period: a monocentric experience. Quant Imaging Med Surg.

[24] Fan L, Liu S, CT, COVID-19 (2020). Chinese experience and recommendations concerning detection, staging and follow-up. Eur Radiol.

[25] Niu Y, Huang S, Zhang H, Li S, Li X, Lv Z (2021). Optimization of imaging parameters in chest CT for COVID-19 patients: an experimental phantom study. Quant Imaging Med Surg.

[26] Zhou Y, Zheng Y, Yang Q, Hu L, Liao J, Li X (2020). Cohort study of chest CT and clinical changes in 29 patients with coronavirus disease 2019 (COVID-19). Eur Radiol.

[27] Zeinali-Rafsanjani B, Alavi A, Lotfi M, Haseli S, Saeedi-Moghadam M, Moradpour M (2023). Is it necessary to define new diagnostic reference levels during pandemics like the Covid19-?. Radiat Phys Chem.

[28] Smith-Bindman R, Lipson J, Marcus R, Kim KP, Mahesh M, Gould R (2009). Radiation dose associated with common computed tomography examinations and the associated lifetime attributable risk of cancer. Arch Intern Med.

[29] Shubayr N, Alashban Y (2022). Estimation of radiation doses and lifetime attributable risk of radiation-induced cancer in the uterus and prostate from abdomen pelvis CT examinations. Front Public Health.

[30] Pierce DA, Shimizu Y, Preston DL, Vaeth M, Mabuchi K. Studies of the mortality of atomic bomb survivors. Report 12, Part I. Cancer: 1950–1990. Radiat Res. 1996;146(1):1–27. 8677290

[31] Jamshidi MH, Karami A, Ordoni J, Bijari S. Estimation of Lifetime attributable risk (LAR) of Cancer Associated with chest computed tomography procedures in children. Front Biomedical Technol. 2023;10(4).

[32] Worldometer. COVID-19 CORONAVIRUS PANDEMIC 2023 [updated November 05, 2023. Available from: https://www.worldometers.info/coronavirus/.

[33] Huang C, Wang Y, Li X, Ren L, Zhao J, Hu Y (2020). Clinical features of patients infected with 2019 novel coronavirus in Wuhan, China. Lancet (London England).

[34] Tiruneh SA, Tesema ZT, Azanaw MM, Angaw DA (2021). The effect of age on the incidence of COVID-19 complications: a systematic review and meta-analysis. Syst Reviews.

[35] Bent C, Lee PS, Shen PY, Bang H, Bobinski M (2015). Clinical scoring system may improve yield of head CT of non-trauma emergency department patients. Emerg Radiol.

[36] Wang X, You JJ (2013). Head CT for nontrauma patients in the emergency department: clinical predictors of abnormal findings. Radiology.

[37] Radiology, ACo. ACR Recommendations for the use of Chest Radiography and Computed Tomography (CT) for Suspected COVID-19 Infection 2020 [updated March 11, 2020. Available from: https://www.acr.org/Advocacy-and-Economics/ACR-Position-Statements/Recommendations-for-Chest-Radiography-and-CT-for-Suspected-COVID19-Infection.

[38] Gao Y, Hu Y, Zhu J, Liu H, Qiu R, Lin Q (2021). The value of repeated CT in monitoring the disease progression in moderate COVID-19 pneumonia: a single-center, retrospective study. Medicine.

[39] Chen L, Wang Q, Wu H, Hu J, Zhang J (2020). REPEAT CHEST CT SCANS IN MODERATE-TO-SEVERE PATIENTS’ MANAGEMENT DURING THE COVID-19 PANDEMIC: OBSERVATIONS FROM A SINGLE CENTRE IN WUHAN, CHINA. Radiat Prot Dosimetry.

[40] Griffey RT, Sodickson A (2009). Cumulative radiation exposure and cancer risk estimates in emergency department patients undergoing repeat or multiple CT. AJR Am J Roentgenol.

[41] Caoili EM, Cohan RH, Ellis JH, Dillman J, Schipper MJ, Francis IR (2009). Medical decision making regarding computed tomographic radiation dose and associated risk: the patient’s perspective. Arch Intern Med.

[42] Barnawi RA, Alrefai WM, Qari F, Aljefri A, Hagi SK, Khafaji M (2018). Doctors’ knowledge of the doses and risks of radiological investigations performed in the emergency department. Saudi Med J.

[43] Hobbs JB, Goldstein N, Lind KE, Elder D, Dodd GD, Borgstede JP (2018). Physician knowledge of Radiation exposure and risk in Medical Imaging. J Am Coll Radiol.

[44] Gimbel RW, Fontelo P, Stephens MB, Olsen CH, Bunt C, Ledford CJ (2013). Radiation exposure and cost influence physician medical image decision making: a randomized controlled trial. Med Care.

[45] Hendee WR, O’Connor MK (2012). Radiation risks of medical imaging: separating fact from fantasy. Radiology.

[46] O’Connor MK (2017). Risk of low-dose radiation and the BEIR VII report: a critical review of what it does and doesn’t say. Phys Medica: PM: Int J Devoted Appl Phys Med Biology: Official J Italian Association Biomedical Phys (AIFB).

